# Increasing inequality in age of death at shared levels of life expectancy: A comparative study of Scotland and England and Wales

**DOI:** 10.1016/j.ssmph.2016.10.001

**Published:** 2016-10-07

**Authors:** Rosie Seaman, Alastair H. Leyland, Frank Popham

**Affiliations:** MRC/CSO Social and Public Health Sciences Unit, University of Glasgow, United Kingdom

**Keywords:** Life expectancy, Lifespan variation, Premature mortality, Mortality inequalities, Public health diffusion

## Abstract

There is a strong negative correlation between increasing life expectancy and decreasing lifespan variation, a measure of inequality. Previous research suggests that countries achieving a high level of life expectancy later in time generally do so with lower lifespan variation than forerunner countries. This may be because they are able to capitalise on lessons already learnt. However, a few countries achieve a high level of life expectancy later in time with higher inequality. Scotland appears to be such a country and presents an interesting case study because it previously experienced lower inequality when reaching the same level of life expectancy as its closest comparator England and Wales. We calculated life expectancy and lifespan variation for Scotland and England and Wales for the years 1950 to 2012, comparing Scotland to England and Wales when it reached the same level of life expectancy later on in time, and assessed the difference in the level of lifespan variation. The lifespan variation difference between the two countries was then decomposed into age-specific components. Analysis was carried out for males and females separately. Since the 1950s Scotland has achieved the same level of life expectancy at least ten years later in time than England and Wales. Initially it did so with lower lifespan variation. Following the 1980s Scotland has been achieving the same level of life expectancy later in time than England and Wales and with higher inequality, particularly for males. Decomposition revealed that higher inequality is partly explained by lower older age mortality rates but primarily by higher premature adult age mortality rates when life expectancy is the same. Existing studies suggest that premature adult mortality rates are strongly associated with the social determinants of health and may be amenable to social and economic policies. So addressing these policy areas may have benefits for both inequality and population health in Scotland.

## Highlights

• Some countries reaching a high level of life expectancy later on in time do so with lower inequality, a few do so with greater inequality.
• Greater inequality in Scotland has largely been driven by relatively high premature mortality alongside relatively low old age mortality causing mortality expansion but not mortality compression.
• Tackling premature mortality will ensure simultaneous improvements in average population health and reductions in inequality.

## 1 Background

There is a strong association between life expectancy and lifespan variation. Life expectancy reflects the average length of life in the population and lifespan variation reflects the variability surrounding the length of life (van Raalte et al., 2011). In any given year, countries with higher life expectancy have lower lifespan variation (Smits and Monden, 2009, Vaupel et al., 2011). Lifespan variation is regarded as a measure of inequality and so there is an association between improving average population health and reducing inequality (Shkolnikov et al., 2011, van Raalte et al., 2011).

### 1.1 Compression and expansion

Reducing the mortality rate at any age will increase life expectancy but improvements in lifespan variation are only achieved if reductions in premature mortality rates are greater than reductions in older age mortality rates. This is because reducing premature mortality rates compresses the age distribution of death while reducing older age mortality rates expands the distribution (Oeppen, 2008, Smits and Monden, 2009, Vaupel et al., 2011). Therefore the desired negative correlation between increasing life expectancy and decreasing lifespan variation is contingent upon more compression than expansion (Shkolnikov et al., 2011). Hence countries reaching the same level of life expectancy at different times can do so with underlying differences in age specific mortality rates, resulting in different levels of lifespan variation (Auger et al., 2014, Nau and Firebaugh, 2012, Shkolnikov et al., 2011).

### 1.2 Lifespan variation differences independent of life expectancy

Given the strong correlation between life expectancy and lifespan variation Smits and Monden (2009) argue that cross national comparisons of lifespan variation should focus on when similar levels of life expectancy are achieved, regardless of when they were achieved. This allows differences in lifespan variation to be studied independently of life expectancy. Differences in lifespan variation at shared life expectancy may reflect contrasting influences on mortality rates between countries. These may include the social determinants of health, public health strategies, and the temporal contexts or stages of epidemiological transition (Omran, 1971, Vallin and Meslé, 2004, Wilmoth and Horiuchi, 1999). Smits and Monden (2009) proposed two hypotheses for how these broad categories might impact overall differences in lifespan variation between countries achieving the same life expectancy at different times; the forerunner hypothesis and the diffusion hypothesis.

### 1.3 The forerunner hypothesis

The forerunner hypothesis proposed that countries reaching a level of life expectancy first do so with lower lifespan variation than those achieving the same life expectancy later. This is because life expectancy pioneers have historically reduced premature mortality rates the most in order to achieve their high levels of life expectancy: which in turn means reduced lifespan variation.

### 1.4 The diffusion hypothesis

On the other hand the diffusion hypothesis suggests that countries reaching the same level of life expectancy later do so with lower lifespan variation than life expectancy pioneers. This is because they have a temporal advantage and can capitalise on the lessons already learnt about reducing mortality rates from the pioneering countries. Or as Vallin and Meslé (2004) state “the most recent arrivals advance more rapidly than the pioneers”. When testing these two hypotheses, using data for 191 countries, Smits and Monden (2009) found that lifespan variation does differ between countries achieving the same level of life expectancy at different times. They found support for the diffusion rather than the forerunner hypothesis: life expectancy pioneers tended to have higher lifespan variation than countries achieving the same life expectancy later on in time.

### 1.5 Laggard countries

Given the apparent lifespan variation advantage countries achieving a certain life expectancy later have, it is perhaps surprising that there are some developed countries achieving the same life expectancy later but with considerably higher levels of inequality. This suggests that there are laggard countries that seem to not be benefiting fully from their temporal advantage.

Laggard countries are perhaps counter-intuitive as the economic cost of reducing premature adult mortality – deaths associated with external causes – tend to be relatively lower than the economic costs of reducing old age mortality – deaths from chronic diseases (Nau and Firebaugh, 2012, Smits and Monden, 2009). Vallin and Meslé (2004) therefore suggest that not all societies are equally prepared to draw on the benefits of earlier innovation or replicate outside practices. We seek to provide some insight into why there are some countries reaching a high level of life expectancy later in time with higher lifespan variation.

The most studied laggard country so far is the USA (Edwards and Tuljapurkar, 2005, Nau and Firebaugh, 2012, Shkolnikov et al., 2011). It has demonstrated higher levels of lifespan variation when achieving the same level of life expectancy as forerunner countries such as Sweden (Smits & Monden, 2009). This can be explained by the fact that the USA has relatively high rates of premature deaths alongside relatively low old age death rates: so at the same time there is large variability in the age at which people are dying prematurely alongside a delaying in the age at which they are expected to die (Shkolnikov et al., 2011, Smits and Monden, 2009). This would suggest that the USA has not fully benefited from the experience of other countries in terms of premature adult age mortality. Yet it has been a leader in terms of reducing older age mortality associated with the temporal advances in modern healthcare practices, for example the treatment of chronic diseases (Vallin & Meslé, 2004).

It is not yet clear if the laggard hypothesis, supported by evidence of higher premature working age mortality alongside lower old age mortality despite a shared level of life expectancy, is applicable to other economically advanced countries within Western Europe: Scotland, a less studied country, provides an opportunity to evaluate this.

### 1.6 Scotland as a case study country

Population health in Scotland is relatively poor by Western European standards (Leon et al., 2003, McCartney et al., 2012b). The people living in Scotland experience the lowest level of life expectancy, with improvements in life expectancy faltering relative to many other Western European countries (McCartney et al., 2012b). Although Scotland's life expectancy is still improving, lifespan variation has been slightly increasing in Scotland over the last two decades with indications of some very recent decreases (Popham and Boyle, 2010, Seaman et al., 2016).

**Fig. 1:**
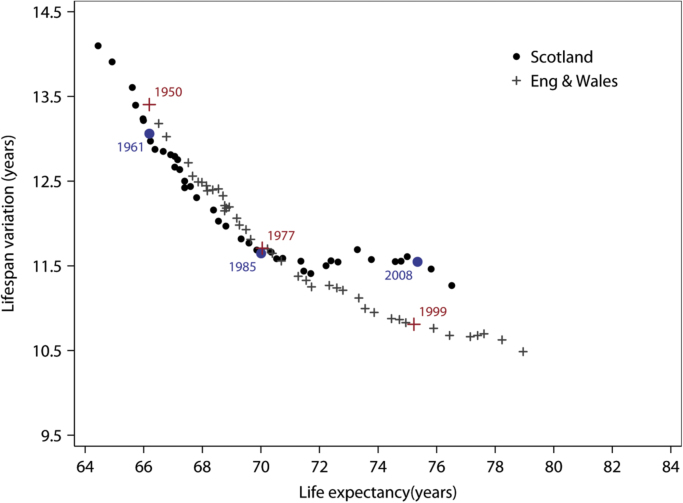
Association between life expectancy and lifespan variation, 1950–2012, males.

**Fig. 2:**
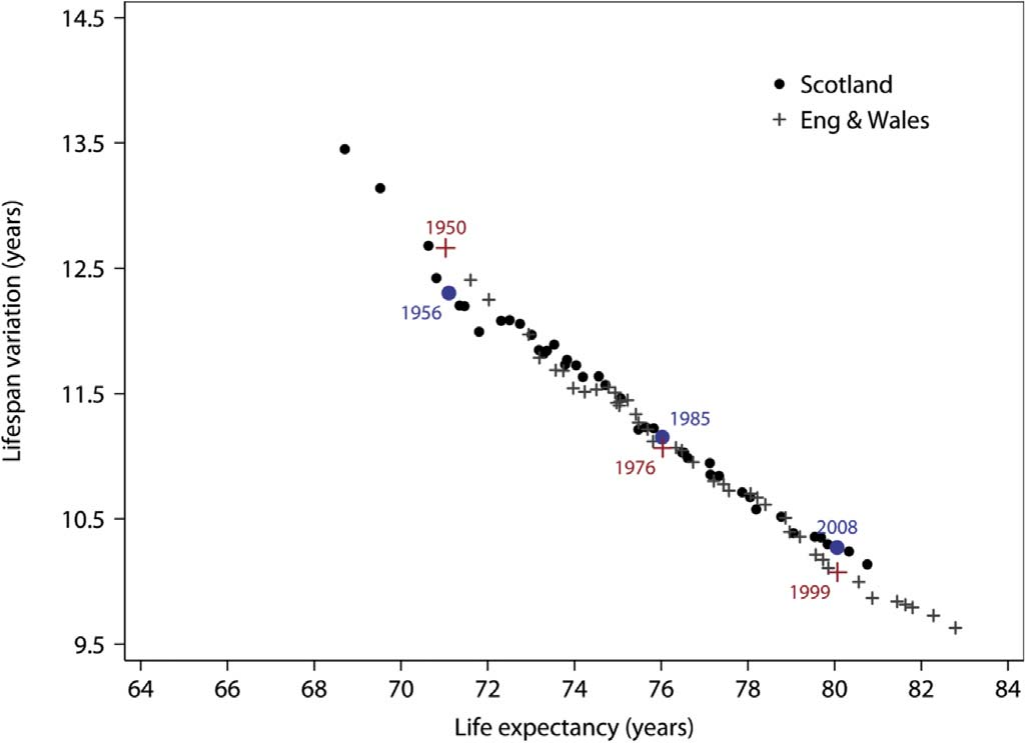
Association between life expectancy and lifespan variation, 1950–2012, females.

When comparing life expectancy trends across Western European countries, McCartney et al. (2012b) found that Scotland's trend in life expectancy had diverged since at least the 1980s around the same period that Seaman et al. (2016) suggested that Scotland's lifespan variation trend diverged. Life expectancy and lifespan variation for Scotland is plotted against its closest comparator England and Wales (see Fig. 1 and Fig. 2 for males and females respectively). England and Wales is a valid comparator country; it is Scotland's closest geographical neighbour; they have experienced the same social and economic developments; and they share a national Government.

Scotland has lagged behind England and Wales, in terms of life expectancy in the same year, since at least 1950. When it did achieve the same level of life expectancy, later in time, it initially did so with lower levels of lifespan variation. Following the 1980s for males and 2000s for females it has reached the same life expectancy, later in time, with higher levels of lifespan variation. Hence Scotland is an important case study country having become a lifespan variation laggard.

It is expected that premature adult mortality in Scotland will be part of the explanation as existing studies show little change or even increasing mortality rates for certain adult age groups over time (Leyland, Dundas, McLoone & Boddy, 2007a; Leyland, 2004; Norman, Boyle, Exeter, Feng & Popham, 2011). For example mortality rates for males aged 15–29 years old increased between 1981 and 2001, while mortality rates for males aged 30–44 years old demonstrated an initial decrease before increasing, meaning that by the end of 2001 the rate had effectively remained unchanged (Leyland et al., 2007a).

Several explanations for Scotland's premature mortality problem have been proposed including; health behaviours, de-industrialisation and deprivation. These explanations were critically evaluated by McCartney, Collins, Walsh, and Batty (2012a) and Walsh, McCartney, Collins, Taulbut, and Batty (2016). They placed emphasis on the upstream explanations that link higher poverty, deprivation and inequality in Scotland to structural changes in employment and the political landscape. Although our study cannot identify the underlying reasons why Scotland appears to have become a lifespan variation laggard it can provide insight into the types of explanations that merit further attention.

## 2 Data and methods

The Human Mortality Database (HMD) provides sex specific lifetables for Scotland and for England and Wales since 1855 and 1841 respectively. The mortality rates from the lifetables for each year from 1949 to 2013 (the latest available year at the time of analysis) were used in this study. This period has also been the focus of much of the research into Scotland's mortality problem (Campbell et al., 2013, McCartney et al., 2012b). Results are reported for males and females separately because their mortality experiences differ.

Age specific mortality rates from the lifetables were used to calculate a three year rolling annual average mortality rate for each age and year from 1950 to 2012. The rolling average mortality rates give equal weight to each year. A rolling average was used to smooth mortality rates that are inherently vulnerable to random fluctuations. The rolling average mortality rates were converted to lifetable probabilities of dying for males and females in Scotland and England and Wales separately. From these lifetable probabilities life expectancy and lifespan variation were calculated (see interactive plot) using methods implemented in a Microsoft Excel spreadsheet provided by the Max Planck Institute for Demographic Research and authored by Shkolnikov and Andreev (2010).

The measure of lifespan variation used was $e_{0}^{†}$. This is the sum of remaining life expectancy at each age, weighted by the proportion of deaths at that age. This was deemed to be the most accessible measure of lifespan variation because it is easily interpreted as the average number of years of life lost per death. A high correlation between several measures of lifespan variation ($e^{†}$, the Gini-coefficient, Theil's index, mean logarithmic deviation, standard deviation and interquartile range) is reported by van Raalte et al. (2011).

We carry out age decomposition using Andreev's stepwise decomposition algorithm within the Microsoft Excel Spreadsheets developed by Shkolnikov and Andreev (2010). Decomposition methods have been widely applied in research to partition the absolute difference in life expectancy or lifespan variation into age components (Auger et al., 2014, Shkolnikov et al., 2011, van Raalte et al., 2014). This approach is appropriate for decomposing, by age, differences in any aggregate measure that is estimated from age-specific mortality rates and accounts for all ages including the oldest open ended age. A detailed description of the ‘general stepwise replacement algorithm’ is available elsewhere Shkolnikov and Andreev (2010).

This study applies stepwise decomposition to calculate the age-specific contributions to any lifespan variation difference between Scotland and England and Wales at shared levels of life expectancy. We also decompose the same level of life expectancy to further illustrate that differences in the age distribution of death can exist even when life expectancy is comparable. This enabled us to establish if the mortality rates contributing to the difference in lifespan variation were also detrimental to Scotland's life expectancy even when life expectancy was similar.

## 3 Results

### 3.1 The association between life expectancy and lifespan variation over time

Fig. 1 and Fig. 2 demonstrate the changing association between life expectancy and lifespan variation for Scotland, for males and females respectively. Scotland has tended to achieve a similar level of life expectancy approximately ten years later than England and Wales. Scotland was previously able to achieve the same level of life expectancy later in time than England and Wales with slightly lower lifespan variation possibly providing some evidence of diffusion. For example Males in England and Wales achieved a life expectancy of 66 years old in 1950 with a lifespan variation of 13.40 years. It took males in Scotland until 1961 to achieve this life expectancy but it did so with a lifespan variation of 13.06 years.

Following 1985, for males, Scotland lost its lifespan variation advantage when it achieved a similar level of life expectancy later in time than England and Wales, suggesting that it had become a laggard. For example, males in Scotland reached a life expectancy of 75 years old in 2008 which males in England and Wales reached in 1999. However Scotland achieved this level of life expectancy with a lifespan variation of 11.55 years which was greater than the 10.81 years of lifespan variation experienced in England and Wales (years highlighted in Fig. 1).

This same change is observed for females but the level of higher lifespan variation, at a shared level of life expectancy, is not as large as seen for males (the scatter plots for females in Scotland diverge less). Females in England and Wales achieved a life expectancy of 71 years old in 1950 with a lifespan variation of 12.66 years. It took Scotland until 1956 to achieve this life expectancy but it did so with a lifespan variation of 12.30 years.

Females in Scotland started to lose any clear lifespan variation following the early 2000s. For example, females in Scotland reached a life expectancy of 80 years old in 2008 which females in England and Wales reached in 1999. However females in Scotland achieved this level of life expectancy with a lifespan variation of 10.27 years which was greater than the 10.07 years of lifespan variation experienced in England and Wales.

### 3.2 Description of decomposition graphs

**Fig. 3:**
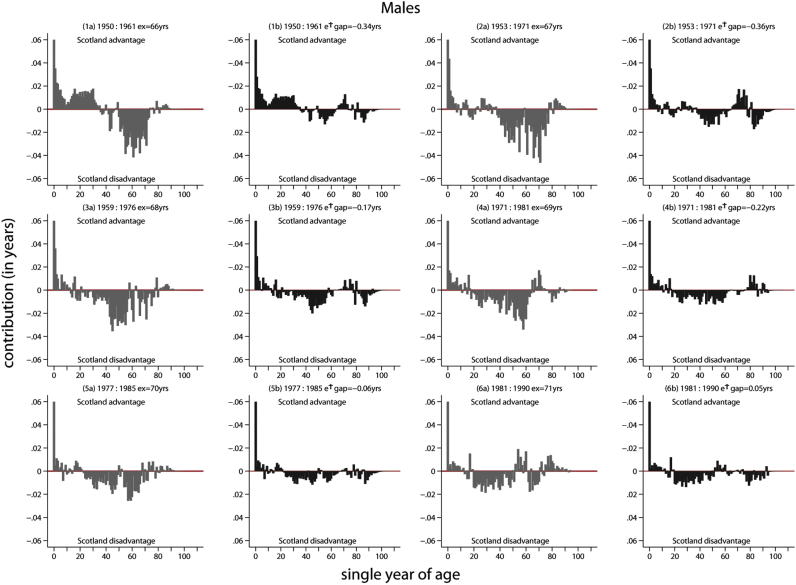
Decomposition results, males. *Year England and Wales achieved life expectancy:* Year Scotland achieved life expectancy. Column (a) is the contributions made to life expectancy at shared level. Column (b) is age contributions to lifespan variation gap at shared levels of life expectancy. First year of life contributions truncated at 0.06 years so as not to dominate the scale during the earliest comparison, full contributions from first year of life reported in Appendix.

**Fig. 4:**
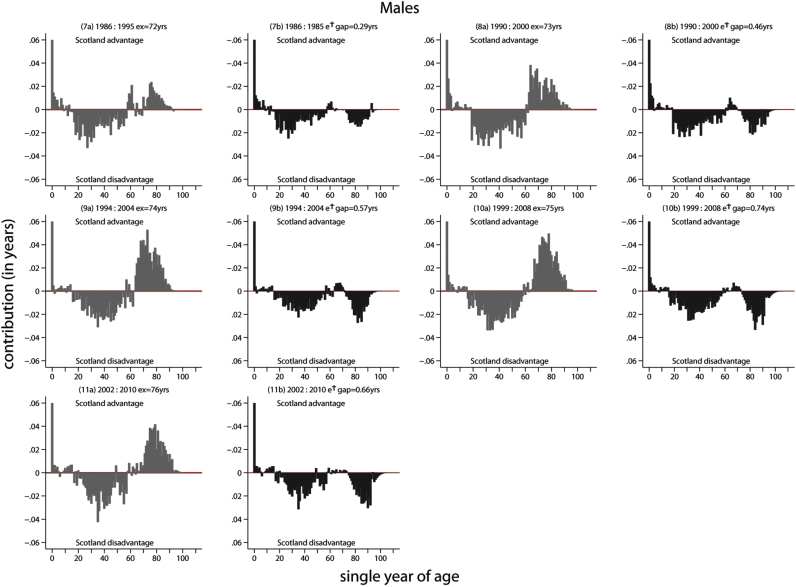
Decomposition results, males. *Year England and Wales achieved life expectancy:* Year Scotland achieved life expectancy. Column (a) is the contributions made to life expectancy at shared level. Column (b) is age contributions to lifespan variation gap at shared levels of life expectancy. First year of life contributions truncated at 0.06 years so as not to dominate the scale during the earliest comparison, full contributions from first year of life reported in Appendix.

**Fig. 5:**
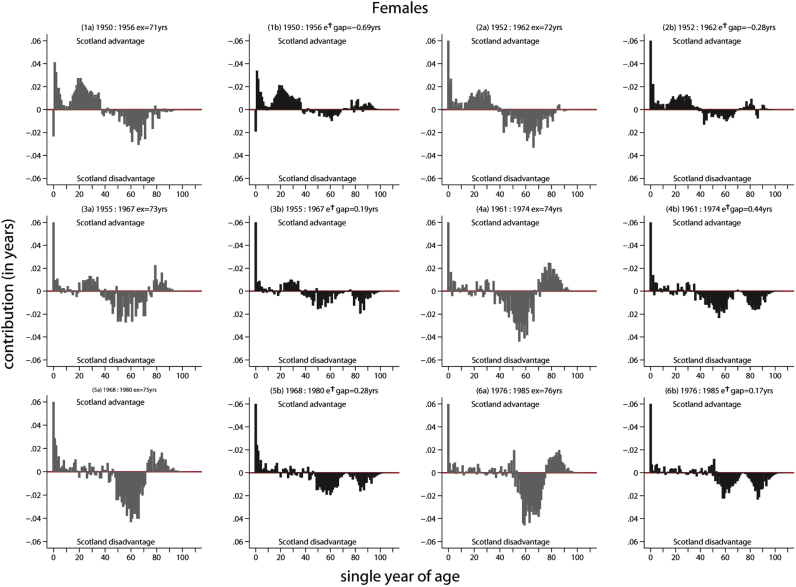
Decomposition results, females. *Year England and Wales achieved life expectancy:* Year Scotland achieved life expectancy. Column (a) is the contributions made to life expectancy at shared level. Column (b) is age contributions to lifespan variation gap at shared levels of life expectancy. First year of life contributions truncated at 0.06 years so as not to dominate the scale during the earliest comparison, full contributions from first year of life reported in Appendix.

**Fig. 6:**
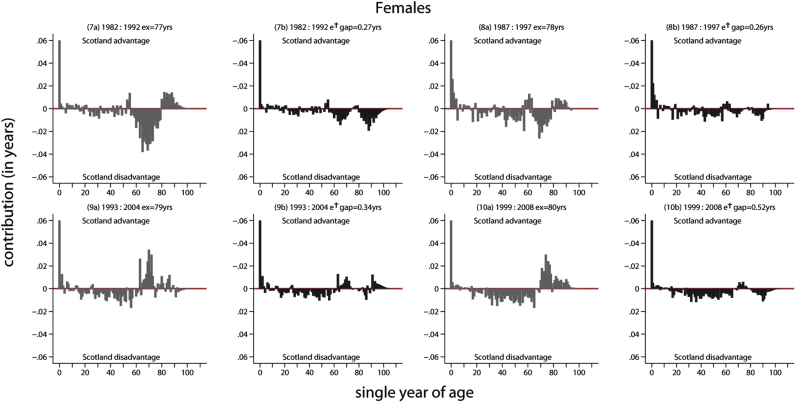
Decomposition results, females. *Year England and Wales achieved life expectancy:* Year Scotland achieved life expectancy. Column (a) is the contributions made to life expectancy at shared level. Column (b) is age contributions to lifespan variation gap at shared levels of life expectancy. First year of life contributions truncated at 0.06 years so as not to dominate the scale during the earliest comparison, full contributions from first year of life reported in Appendix. (For interpretation of the references to color in this figure, the reader is referred to the web version of this article.).

The decomposition results are reported in Fig. 3 and Fig. 4 for males. The decomposition results for females are reported in Fig. 5 and Fig. 6.

For each decomposition there are two graphs. The first graph in column (a) shows the contribution of age differences in mortality rates to life expectancy when a shared level of life expectancy was achieved (grey spikes). This is to demonstrate that age specific mortality can differ between countries even when life expectancy is comparable (Auger et al., 2014). If there were no differences in the age specific mortality rates, at the same level of life expectancy, there would be no spikes as there is zero difference at every age.

The second graph in column (b) shows the age contribution from differences in age mortality rates to any lifespan variation gap when a shared level of life expectancy was achieved (black spikes).

Comparing across column (a) and column (b) establishes if the differences in the age specific mortality rates were to Scotland's life expectancy advantage (above the red line) or life expectancy disadvantage (below the red line) while simultaneously being to Scotland's lifespan variation advantage (above the red line) or to Scotland's lifespan variation disadvantage (below the red line).

The title of each graph states the years that are being compared and the level of life expectancy or lifespan variation difference that the decomposition results refer to. The earlier year always refers to when England and Wales achieved this life expectancy and the later year always refers to when Scotland achieved this life expectancy.

There are some distinct differences in the age pattern of mortality between the two countries revealing inequalities underneath shared levels of life expectancy. Scotland reached the same level of life expectancy as England and Wales with lower mortality rates across some ages and higher mortality rates across others. We broadly describe the inequalities in the age patterns in terms of infant/early life mortality (age 0–15 years old), premature adult mortality (16–64 years old) and older age mortality (65+ years old).

### 3.3 Decomposition at shared levels of life expectancy

Scotland has always reached the same level of life expectancy later in time with lower or similar mortality rates across infant/early life ages (0–15 years old). This pattern is found for males (Fig. 3 and Fig. 4) and for females (Fig. 5 and Fig. 6). The advantage this gave Scotland's life expectancy and lifespan variation is particularly evident for males at a shared life expectancy of 66 years old. Scotland achieved this in 1961 with a lifespan variation of 13.06 years. England and Wales achieved this in 1950 but with a lifespan variation of 13.40 years. Lower mortality rates across infant/early life ages therefore simultaneously improved average population health and reduced inequality because they compressed the age distribution.

Over time the gains made from lower infant/early life mortality rates in Scotland diminish. The much lower mortality rate during the first year of life in Scotland in 1971 compared to mortality during the first year of life in England and Wales in 1953 contributed 0.57 years of life expectancy advantage to Scotland. The slightly lower mortality rate during the first year of life in Scotland in 2000 compared to England and Wales in 1994 contributed 0.09 years of life expectancy advantage. This shows that mortality during the first year of life became more comparable between Scotland and England and Wales at a shared level of life expectancy and that Scotland's higher lifespan variation was not primarily due to higher mortality during the first year of life.

Scotland had lower old age mortality rates at a shared level of life expectancy. Lower mortality rates at older ages do not simultaneously increase life expectancy and decrease lifespan variation. This is because they can contribute to mortality expansion. The contributions from lower old age mortality are to Scotland's life expectancy advantage (column (a) grey spikes above the red line) but to its lifespan variation disadvantage (column (b) black spikes below the red line).

This contributed to Scotland achieving a similar life expectancy later in time but more unequally than England and Wales. For example in 2008 Scotland achieved a life expectancy of 75 years old with a lifespan variation of 11.55 years. England and Wales achieved this level of life expectancy in 1999 but with a lifespan variation of 10.81 years.

However, the main contribution to Scotland's greater lifespan variation at the same level of life expectancy was some higher mortality rates across ages that are considered to be premature adult deaths (16–64 years old).

Higher mortality rates across ages 16–64 years old in Scotland, when it achieved the same level of life expectancy as England and Wales but later in time, have always been detrimental to its life expectancy and lifespan variation (contributions always below the red line). This age pattern becomes relatively more predominant over time. It is perhaps most evident when the lifespan variation gap was greatest (0.74 years) at a shared life expectancy of 75 years old: in Scotland this was achieved in 2008 but had already been achieved by England and Wales in 1999.

## 4 Discussion

### 4.1 Summary of results

We found that Scotland has achieved the same level of life expectancy as England and Wales around ten years later in time. Initially it did this with lower lifespan variation. It lost this advantage following the 1980s. Although higher lifespan variation was partly driven by expansion caused by falling mortality at older ages, higher premature working age mortality in Scotland explained much of the higher lifespan variation. This demonstrates that Scotland has become a laggard because of its relatively high adult premature mortality rates which fail to offset the relatively low old age mortality rates.

### 4.2 Strengths and limitations

Our analyses used the most robust, up-to-date data available from the Human Mortality Database (Jasilionis, 2015, Philipov, 2015). The annual mortality rates available from the HMD were used to calculate a three- year rolling average to account for any inherent random fluctuations in the annual mortality rates.

An advantage of our study is the significant time period it covers. We capture the time period when lifespan variation was lower at shared levels of life expectancy and when lifespan variation changed to be higher at shared levels of life expectancy.

The comparator country, England and Wales, was chosen because it shares social and economic contexts as well as a national Government with Scotland and is its closest geographical neighbour. These countries also have had a longstanding, formal commitment to reducing mortality inequalities (Department of HealthDepartment of HealthDepartment of Health, 1999, Department of Health, 2013, The Scottish Government, 2008). The different mortality experience of these countries has been the focus of much research (Campbell et al., 2013, Carstairs and Morris, 1989). Our findings provide further evidence demonstrating the timing of mortality change in Scotland, notably since the 1980s (Campbell et al., 2013; Leyland, Dundas, McLoone & Boddy, 2007b; Norman et al., 2011). It is important that there is consensus across studies around the timing of change if we are to begin to evaluate the reasons for change.

### 4.3 Comparisons with existing studies

This is not the first study to identify a country which provides support for a laggard hypothesis in terms of the age characteristics of mortality decline. The USA is perhaps one of the most widely cited examples (Shkolnikov et al., 2011, Smits and Monden, 2009). Comparative studies, such as ours, are important if we are to understand different levels of inequality (lifespan variation) relative to neighbouring countries at the same level of average population health (life expectancy). We have demonstrated that the changing level of lifespan variation experienced in Scotland when it achieved the same level of life expectancy, later in time, as England and Wales makes it a valuable case study. Case study countries such as Scotland and the USA can help to understand why age patterns of mortality have not been declining uniformly across comparable countries.

Higher lifespan variation in Scotland, when arriving at a particular life expectancy later in time than England and Wales, was partly explained by lower old age mortality rates. This caused mortality expansion which adds uncertainty by delaying the age of death. These lower old age mortality rates suggest that Scotland may have experienced a temporal advantage across these ages but this temporal advantage did not translate into lower mortality rates across all ages. So when achieving the same life expectancy Scotland seems to have benefitted temporally in terms of old age mortality and younger age mortality but not premature adult mortality. The USA demonstrated a similar lack of mortality compression due to higher adult premature mortality. This was coupled with higher levels of expansion associated with lower old age mortality rates compared to England and Wales when both countries had roughly the same life expectancy (Shkolnikov et al., 2011). Unlike Scotland, the USA also had relatively worse young age mortality rates further adding to its higher level of lifespan variation (Shkolnikov et al., 2011). Like Scotland, the USA has become relatively more unequal than other countries in recent decades and when achieving the same life expectancy (Smits & Monden, 2009). So while not exactly the same, the USA and Scotland have similar problems with high premature mortality that may suggest underlying root causes.

Comparative research strongly suggests that the root causes of premature mortality in the USA relate to its poor performance in tackling the social determinants of health (Woolf & Aron, 2013) A recent extensive review of mortality in Scotland also suggests that the lasting effects of deindustrialisation, deprivation and higher inequality have resulted in it experiencing an excess of 5000 deaths per year (Walsh et al., 2016). These are all important social determinants of health and mortality which social and economic policies can alleviate (Bambra, 2011, Coburn, 2000, McCartney et al., 2013).

Considering this, along with the findings in this paper, the mechanisms linking deprivation and premature adult age deaths, could reside with Scotland's inability to reduce and effectively manage social and economic risk particularly for the most vulnerable members of society. Understanding how social and economic policies impact population level mortality has implications for countries across Western Europe but is particularly important for Scotland as it contains some of the poorest communities experiencing some of the worst health outcomes in the UK (McCartney et al., 2012b, The poverty Site, 2016).

Future research should be aimed at identifying whether the level of relative inequality (measured as the level of lifespan variation independent of life expectancy) observed at the population level in Scotland is present across all socioeconomic groups or if the burden of high lifespan variation rests disproportionately on the most deprived because of their higher risk of premature death.

## 5 Conclusions

The results here add to the body of evidence demonstrating that there are countries which lag behind in terms of lifespan variation even at the same level of life expectancy by exploring Scotland. While Scotland seems to have benefited from its temporal advantage in terms of infant/childhood deaths and older age deaths it has failed to benefit when it comes to premature working adult age mortality. This causes a lack of mortality compression alongside mortality expansion and means there is more inequality at the same level of life expectancy. Without tackling premature working age mortality Scotland will not be able to simultaneously achieve improvements in average population health and reductions in inequality.

## Figures and Tables

**Table A1:** Full contributions from age 0 only – males. (Fig. 3 shows contributions truncated to 0.06 years).

Shared ex (approx.)	Year achieved (*England*: Scotland)	Contribution from age 0 at shared life expectancy (years)	Contribution from age 0 to lifespan variation gap (years)
66	*1950*:1961	0.20	−0.16
67	*1953*:1971	0.57	−0.46
68	*1959*:1976	0.50	−0.41
69	*1971*:1981	0.47	−0.39
70	*1977*:1985	0.35	−0.29
71	*1981*:1990	0.25	−0.21
72	*1986*:1995	0.30	−0.25
73	*1990*:2000	0.23	−0.19
74	*1994*:2004	0.09	−0.08
75	*1999*:2008	0.11	−0.09
76	*2002*:2010	0.12	−0.10

The scale of the y axis in the decomposition figures was truncated at 0.06 years as contributions from mortality during the first year of life dominated the scale for the earliest years being compared. These are the full contributions made from the mortality rate difference during the first year of life.

**Table A2:** Full contributions from age 0 only – females. (Fig. 4 shows contributions truncated to 0.06 years).

Shared ex (approx.)	Year achieved (*England*: Scotland)	Contribution from age 0 at shared life expectancy (years)	Contribution from age 0 to lifespan variation gap (years)
71	*1950*:1956	−0.02^a^	0.02^a^
72	*1952*:1962	0.16	−0.11
73	*1955*:1967	0.10	−0.13
74	*1961*:1974	0.27	−0.22
75	*1968*:1980	0.35	−0.30
76	*1976*:1985	0.31	−0.26
77	*1982*:1992	0.25	−0.24
78	*1987*:1997	0.24	−0.20
79	*1993*:2004	0.10	−0.07
80	*1999*:2008	0.10	−0.09

The scale of the y axis in the decomposition figures was truncated at 0.06 years as contributions from mortality during the first year of life dominated the scale for the earliest years being compared. These are the full contributions made from the mortality rate difference during the first year of life.

a Scotland had a very slightly higher infant mortality rate (0.02657) than England and Wales (0.02624) meaning the mortality difference at age 0 made a small, detrimental contribution to Scotland's lifespan variation.

## Appendix A

See Table A1, Table A2.
